# Supplementary material for: Validation of MRI-Based Models to Predict MGMT Promoter Methylation in Gliomas: BraTS 2021 Radiogenomics Challenge
Source: Cancers (Basel). 2022 Oct 3;14(19):4827. doi: 10.3390/cancers14194827 (PMC9562637; doi:10.3390/cancers14194827)
Supplement: Supplementary file 1 [file cancers-14-04827-s001.zip › cancers-1886618-supplementary.pdf]

Supplementary Materials

Statistical analysis

The Kolmogorov–Smirnov test assessed the normality of all the continuous variables to decide whether we should perform parametric or non-parametric test for comparison of the variables. For the analysis of patient characteristics, Pearson's χ^2 test and Student's *t*-test were performed for categorical variables and continuous variables, respectively. For the analysis of proportions compared to chance level, one proportion z-test was performed. For the survival analysis, Kaplan-Meier method with log-rank test was performed. For the analysis of diagnostic performance, receiver operating characteristic (ROC) analysis was performed. All statistical analyses were performed by using MedCalc statistical software, version 20.015 (MedCalc, Mariakerke, Belgium); $p < 0.05$ was considered significant.

Genetic diagnosis for MGMT promoter methylation

The *MGMT* promoter methylation status of the Brain Tumor Segmentation (BraTS) 2021 dataset was determined at each of the host institutions based on various techniques, including pyrosequencing, and next generation quantitative bisulfite sequencing of promoter CpG sites. Sufficient tumor tissue collected at time of surgery was required for both approaches. For the pyrosequencing approach, the genomic DNA was initially extracted from 5ml tissue sections of formalin-fixed paraffin embedded (FFPE) tissue samples. DNA was further cleaned and purified. The DNA concentration, protein to nucleic acid ratio, and DNA to RNA ratio for purity were assessed by spectrophotometer. Approximately 500–1000ng total DNA was subjected to bisulfite conversion using the EPiTect Bisulfite Kit. A total of 50–100 ng bisulfite-treated DNA was carried on for PCR using F-primer and R-primer. Pyrosequencing methylation assay was then conducted using the sequencing primer on the PyroMark Q96ID pyrosequencer. The Pyromark CpG *MGMT* kit detected the average level of methylation on CpG 74–81 sites located in the *MGMT* gene. A cytosine not followed by a guanine served as an internal control for completion of bisulfite conversion. The percent methylation above 10% was interpreted as positive. A sample below 10% methylation was interpreted as negative. For the latter approach, a total of 17 *MGMT* promoter CpG sites were amplified by nested polymerase chain reaction (PCR) using a bisulfite treated DNA template. Quantitative PCR was performed for each CpG site to determine its methylation status. A result of 2% or more methylated CpG sites in the *MGMT* promoter (out of 17 total sites) was considered a positive result [1]. Similarly, for our in-house SNUH dataset, *MGMT*-specific polymerase chain reaction using a methylation EZ Kit (https://www. qiagen.com/us/listpages/ez1-kits/) was used to evaluate the methylation status of the *MGMT* promoter. The ground truth labels for *MGMT* methylation were given as either 0 or 1 for unmethylated or methylated status, respectively. 273 of the 400 patients have been previously reported[2]. This prior article dealt with development of *IDH* prediction and used perfusion-weighted MRI whereas in this manuscript we report and developed the prediction model for the *MGMT* methylation status using mpMRI.

Model Training

The whole training, validation and testing procedures were conducted on a dedicated workstation equipped with four NVIDIA RTX 3090 (NVIDIA, Santa Clara, USA) graphical processing units. The training and inference process were implemented with PyTorch v1.7.0. Adam optimizer with an initial learning rate of 0.001 and mini-batch size of 8 were used for training the model. The learning rate was decayed by a factor of 0.5 if the validation loss is not improved (i.e. decreased) for consecutive 3 epochs. Early stopping was employed to improve generalizability of the model, so the number of training epochs was determined by the highest validation accuracy during training. Specifically, we set the total possible training epochs to 100, where the training stopped if there were no improvement in the validation accuracy for 15 epochs. It should be noted that the final trained model is considered as the model at the best validation accuracy epoch, not at the last training epoch. We used the term training/validation, and internal/external test appropriately as the equivalent terms as the training/tuning, and internal/ external validation, respectively[3].

Convolutional neural network architectures

For SEResNet, we used SEResNet-50, which has "Squeeze-and-Excitation (SE)" module that adaptively recalibrates channel-wise feature responses by explicitly modeling inter-dependency between channels, added to ResNet module[4,5], winning the ImageNet Large Scale Visual Recognition Challenge (ILSVRC) 2017 challenge. For SEResNext, we used SEResNext-50, which added the SE module to ResNext, the 2nd place solution of ILSVRC 2016. ResNext bottleneck, or Aggregated Residual Transformations, have introduced the 1x1 convolution [6], inheriting the strategy of "Split-Transform-Merge"[7] in network engineering. For DenseNet, we used DenseNet-121, which consists of 4 DenseBlocks, densely connecting each layer to every other layer. Compared to residual block, which "adds" the feature maps, in ResNet[4], DenseBlock "concatenates" the feature maps to alleviate the vanishing-gradient problem, strengthen feature

propagation, encourage feature reuse, and substantially reduce the number of parameters[8]. For EfficientNet, we used EfficientNet –B0, which scales depth, width, and resolution, simultaneously to optimize the model, using "compound coefficient"[9]. The B0 model has 15 Mobile Inverted Residual Bottleneck Block (MBConvBlock). The model specifications are summarized in separate supplementary files for each of the architecture, which are the implementations obtained from MONAI package[10].

Model performances analysis submitted to BraTS challenge

Interestingly, when comparing the submitted model performance (i.e. AUROC in private leader board (LB), or a separate test set, which is not publicly released until the end of the challenge**),** and the distribution of random guessing (i.e. randomly generated prediction), there was no difference between the distributions (Supplementary Fig. S3a and S3b). In other words, randomly generated prediction probability could make the similar distribution of AUROCs that submitted to the challenge[11]. In addition, when comparing ranks and AUROCs of public and private LB, top rankers (or AUROCs) in private LB was not concordant, and vice versa[12] (Supplementary Fig. S4a and S4b), which means none of the submitted models show good ranks and AUROCs in both the public and private LB, but only in either one of public or private LB. In other words, the submitted models including 1st place solution would not show the same AUROC in other external validation set, or sufficiently generalized, which is actually proven in our study. These analyses are totally adopted from the BraTS 2021 challenge forum[11,12].

In BraTS 2021 challenge, given a large number of data (*n*=585) with multiparametric inputs, all the participants including 1st place team (AUROC, 0.62) could not discover a reliable MR imaging features that correlates to *MGMT* methylation in gliomas. The test AUROC and accuracy of the 1st place solution of BraTS 2021 challenge was 56.2%, and 54.8%, respectively, when externally validated on SNUH dataset, which is nearly chance level (50%) and not sufficient performance for clinical application. Even combining an additional dataset from our institution, which comprises the largest (*n*=985) dataset in total, the best model among total 420 developed models showed AUROC, and accuracy of 64.5% (mean, 51.7±7.7%) and 55.6% (mean, 51.9±3.4%) in test set, using merged dataset, and 80.2% (337/420), and 60.0% (252/420) of the 420 developed models showed no significant difference with chance level (50%) in terms of test accuracy and test AUROC, respectively. Thus, using a larger dataset of conventional MRI sequences only, could not significantly improve the diagnostic performance, which suggests, at least, the additional information or other advanced algorithms are definitely required for improvement.

Captions for Supplementary Figures

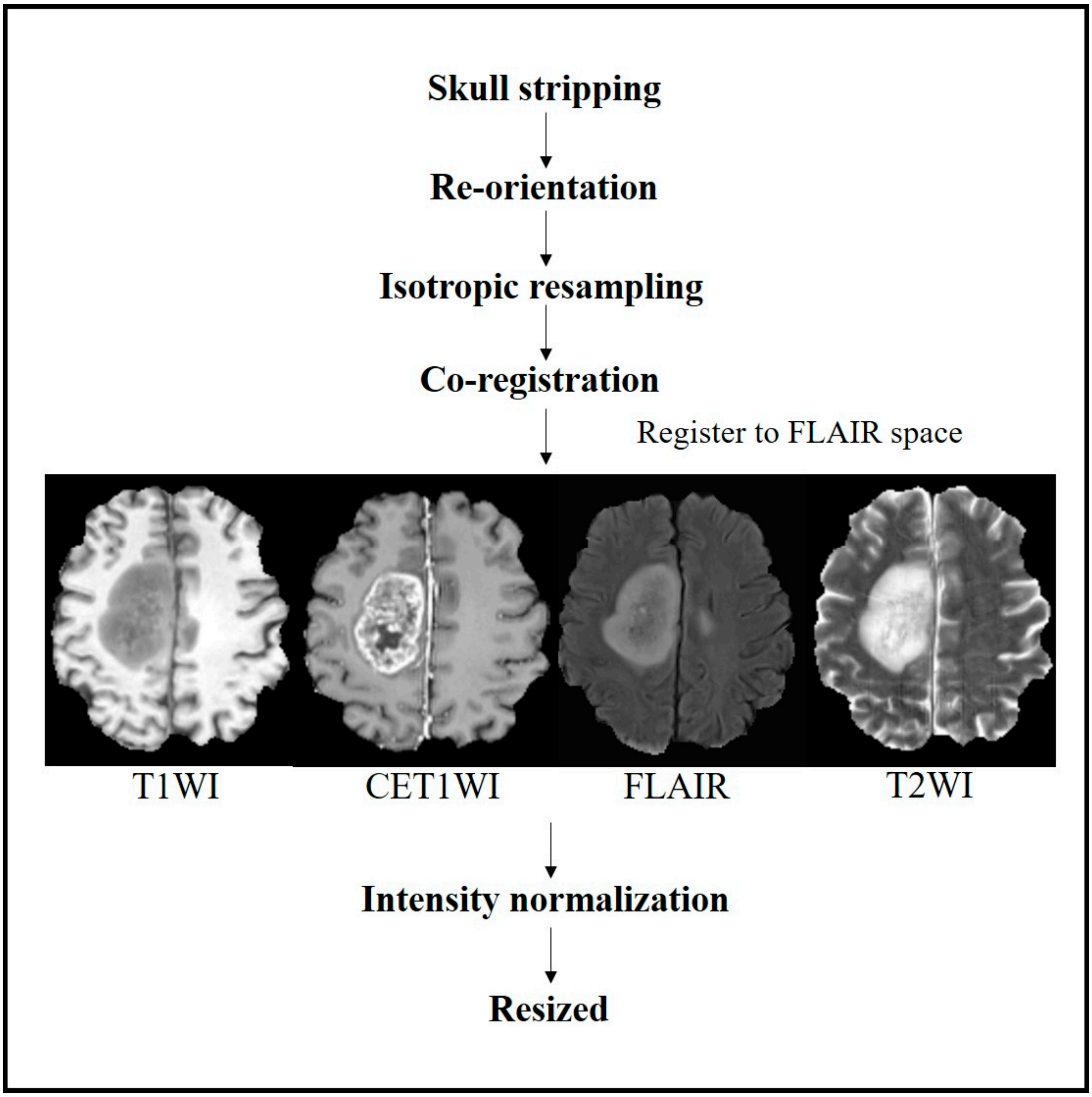

Figure S1. MRI preprocessing pipeline. All 3D volume multiparametric MR images, including T1w, T2w, T1wCE, and T2 FLAIR sequences, were skull stripped, centered, reoriented, resampled to isotropic 1mm, rigidly registered, rescaled intensity to [0,1], and finally resized to the same size. All the preprocessing pipeline was uniformly applied to both BraTS and SNUH dataset. Abbreviations: T1w, T1-weighted imaging; T2w, T2-weighted imaging; T1wCE, contrast-enhanced T1-weighted imaging; FLAIR, fluid attenuated inversion recovery.

(a)

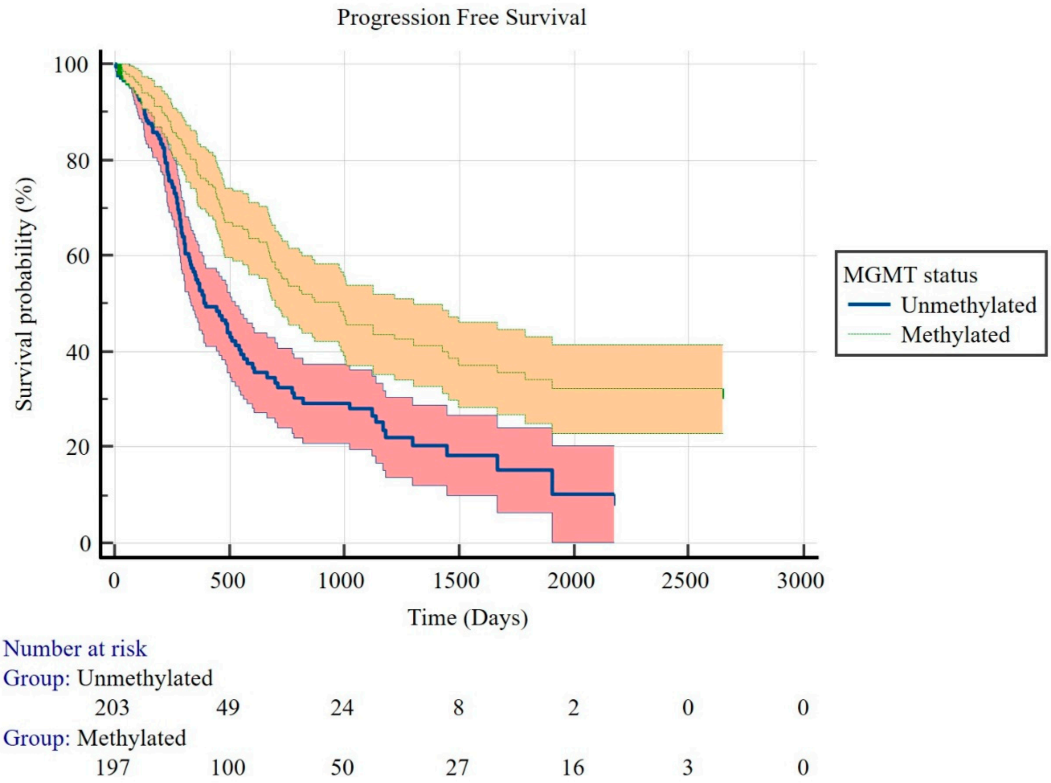
Progression Free Survival
Survival probability (%)
100
80
60
40
20
0
0
500
1000
1500
2000
2500
3000
Time (Days)
MGMT status
Unmethylated
Methylated
Number at risk
Group: Unmethylated
203 49 24 8 2 0 0
Group: Methylated
197 100 50 27 16 3 0

(b)

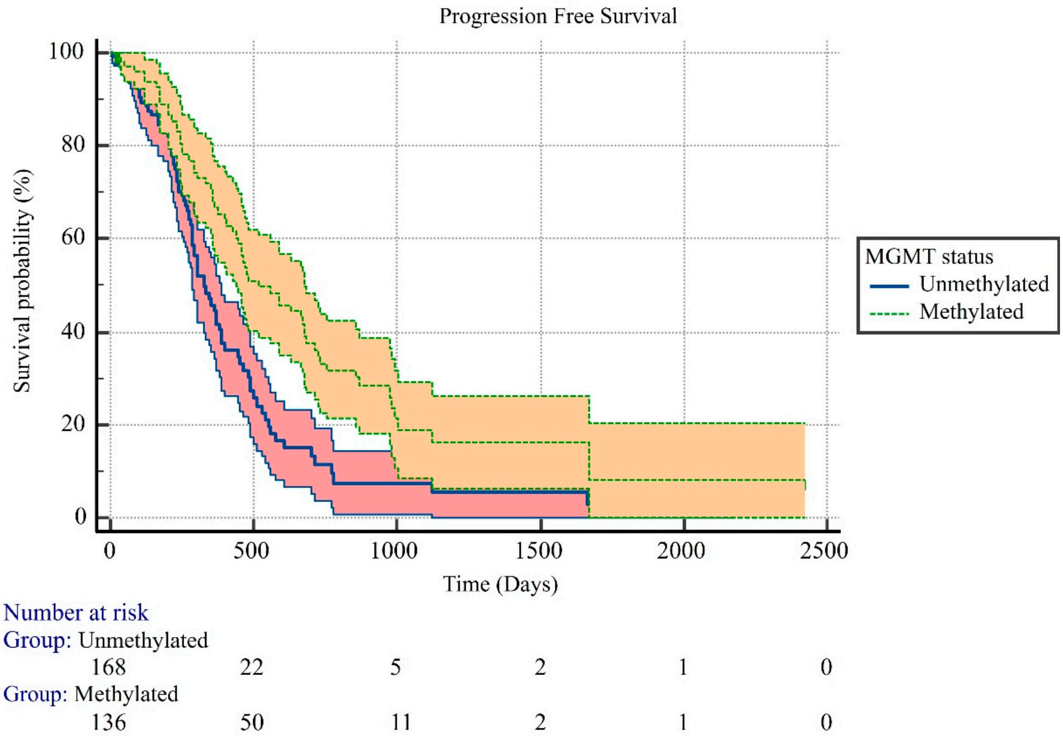
Progression Free Survival
Survival probability (%)
100
80
60
40
20
0
0
500
1000
1500
2000
2500
Time (Days)
MGMT status
Unmethylated
Methylated
Number at risk
Group: Unmethylated
168 22 5 2 1 0
Group: Methylated
136 50 11 2 1 0

(c)

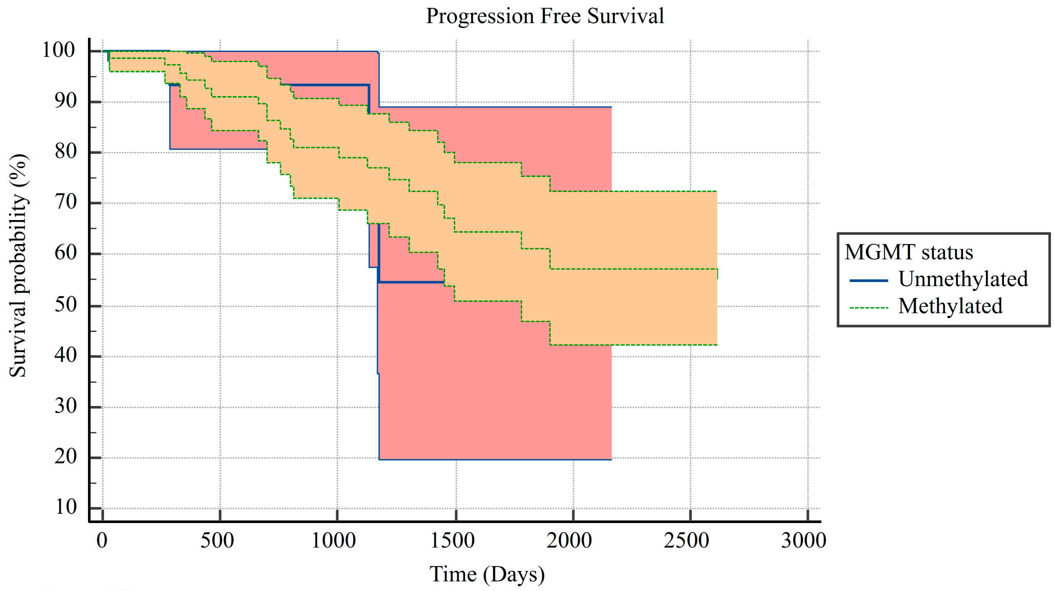

Number at risk
Group: Unmethylated

35	22	16	5	2	0	0

Group: Methylated

61	47	32	19	11	2	0

Figure S2. Progression free survival (PFS) curves according to the methylation status of *MGMT* promotor in (a) SNUH dataset (*n*=400); (b) IDH-wildtype (n=304); and (c) IDH-mutant (n=96) subgroups. Note that PFS was significantly longer in methylated than unmethylated group: (a) median, 396 vs 974 days(p<0.0001) in all diffuse gliomas (n=400); and (b) median, 328 vs 514 days (p=0.0001) in IDH-wildtype subgroup. However, PFS showed no difference between MGMT methylated and unmethylated group in (c) IDH-mutant subgroup: mean, 1949 vs 1650 days (p=0.871).

(a) **(b)**

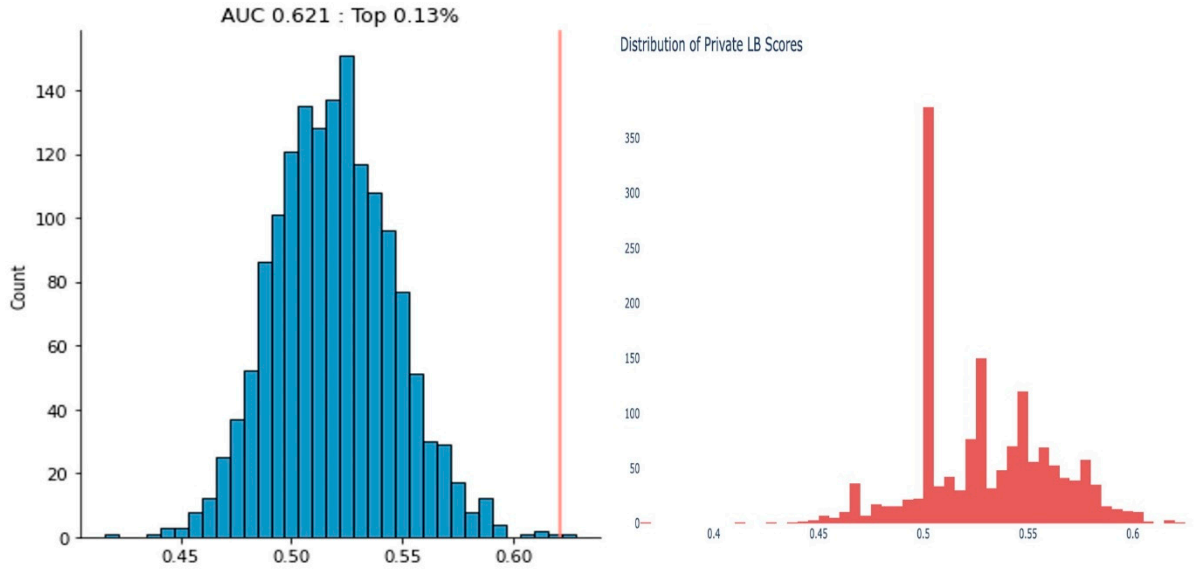

Figure S3. The distribution of (a) randomly generated prediction AUROC scores, and (b) private LB scores. Note that there is no difference between the two distributions, and AUROC of 1st place solution (0.621) can be reached in top 0.13% in the distribution of the randomly generated predictions (Adopted from the BraTS 2021 challenge forum[11]). Abbreviations: LB, leader board of the BraTS 2021 challenge; AUROC, area under the receiver-operating characteristic curve.

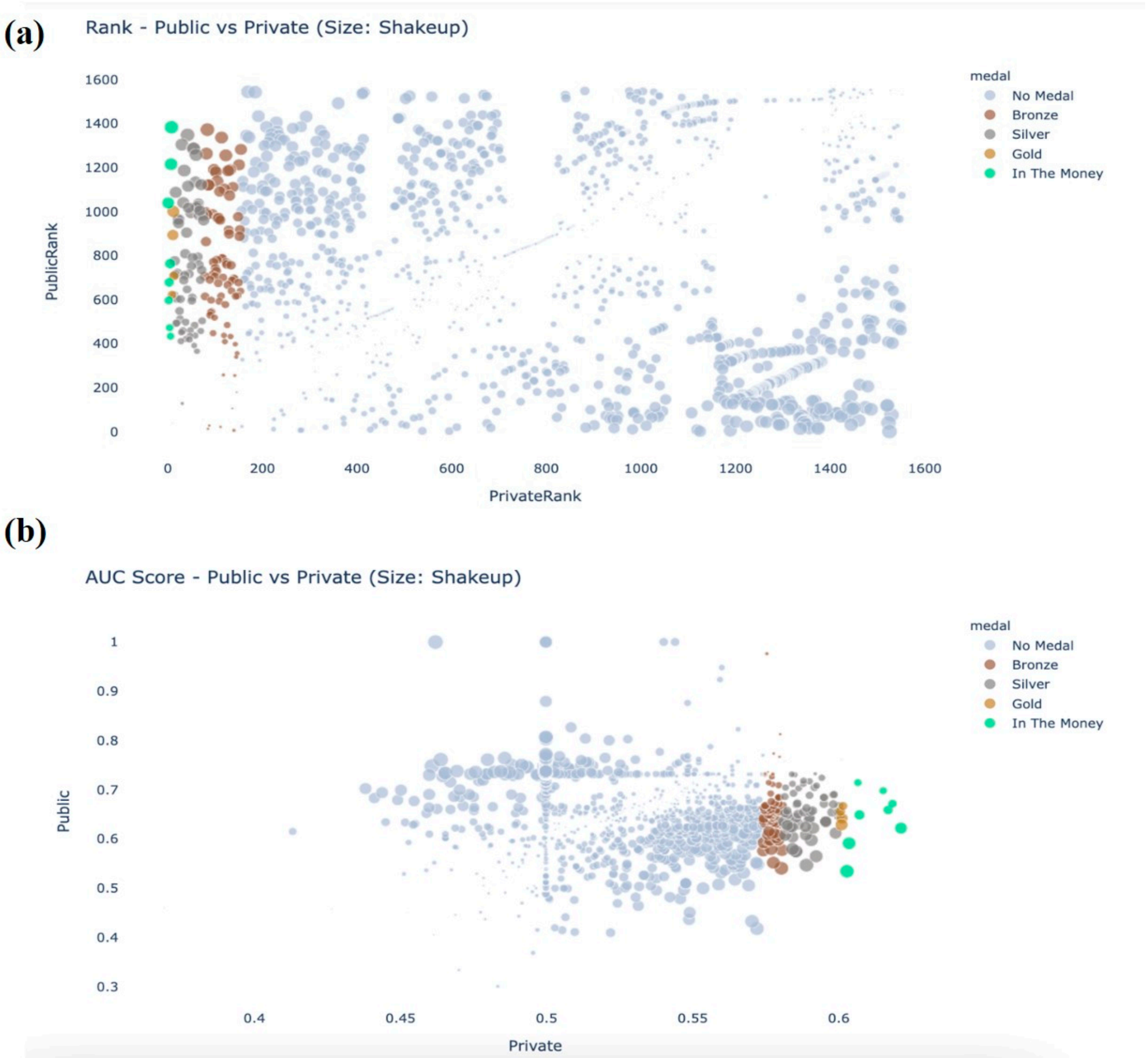

Figure S4. The shakeup plots of (a) ranks and (b) AUROCs obtained from public vs private leader board of the BraTS 2021 challenge. Note that none of the submitted models show good ranks and AUROCs in both the public and private LB, but only in either one of public or private LB (Adopted from the BraTS 2021 challenge forum[12]). Abbreviations: AUC scores, or AUROC, area under the receiver-operating characteristic curve.

Table S1. MRI scan parameters.

	SIGNA EXCITE (GE)	SIGNA HDXT (GE)	DISCOVERY MR750W (GE)	VERIO (SIEMENS)	SKYRA (Siemens)
Field strength (T)	3.0	1.5	3.0	3.0	3.0
Head coil channel	8	8	32	32	64
T1WI					
TR(ms)	466.7	10.4	8.4	1420	1600
TE(ms)	11.0	4.7	3.2	1.9	2.8
FA(°)	69	20	12	9	9
NEX	1	1	1	1	1
Matrix	320x192	240x240	256x230	256x232	256x248
Section thickness(mm)	5	1	1	1	1
Intersection gap(mm)	1	0	0	0	0
FOV(mm)	220x220	240x240	256x256	249x249	249x249
T2WI					
TR(ms)	5000	4850	5124.5	5100	5100

TE(ms)	131.1	121.9	92.6	89	89
FA(°)	90.0	90	142	16	150
NEX	2	2	2	3	3
Matrix	448x256	448x256	448x448	640x384	640x324
Section thickness(mm)	5	5	5	5	5
Intersection gap(mm)	1	1	1	1	1
FOV(mm)	220x220	220x220	220x220	199x220	185x220
CET1WI					
TR(ms)	1500	1500	1500	1600	1600
TE(ms)	40	40	29.3	30	30
FA(°)	35	35	60	90	90
NEX	1	1	1	1	1
Matrix	128x128	128x128	100x100	128x128	128x128
Section thickness(mm)	5	5	5	6	6
Intersection gap(mm)	1	1	1.5	0.9	0.9
FOV(mm)	220x220	240x240	240x240	240x240	240x240
Scan time	1 min 30 sec	1 min 30 sec	1 min 30 sec	1 min 44 sec	1 min 44 sec

Abbreviations: MRI=magnetic resonance imaging; T1WI=T1-weighted imaging; T2WI=T2-weighted imaging; TR=repetition time; TE=echo time; FA=flip angle; NEX=number of excitations; FOV=field of view; DSC-MRI=dynamic susceptibility contrast (DSC) perfusion MRI.

References

1. Baid, U.; Ghodasara, S.; Mohan, S.; Bilello, M.; Calabrese, E.; Colak, E.; Farahani, K.; Kalpathy-Cramer, J.; Kitamura, F.C.; Pati, S.; et al. The RSNA-ASNR-MICCAI BraTS 2021 Benchmark on Brain Tumor Segmentation and Radiogenomic Classification. *arXiv* **2021**, arXiv:2107.02314.
2. Choi, K.S.; Choi, S.H.; Jeong, B. Prediction of IDH genotype in gliomas with dynamic susceptibility contrast perfusion MR imaging using an explainable recurrent neural network. *Neuro-Oncol.* **2019**, *21*, 1197–1209. https://doi.org/10.1093/neuonc/noz095.
3. Moons, K.G.; Altman, D.G.; Reitsma, J.B.; Ioannidis, J.P.; Macaskill, P.; Steyerberg, E.W.; Vickers, A.J.; Ransohoff, D.F.; Collins, G.S. Transparent Reporting of a multivariable prediction model for Individual Prognosis or Diagnosis (TRIPOD): Explanation and elaboration. *Ann. Intern. Med.* **2015**, *162*, W1–W73. https://doi.org/10.7326/m14-0698.
4. He, K.; Zhang, X.; Ren, S.; Sun, J. *Deep Residual Learning for Image Recognition*, 2016 IEEE Conference on Computer Vision and Pattern Recognition (CVPR), 2016, 770-778. doi: 10.1109/CVPR.2016.90.
5. Hu, J.; Shen, L.; Sun, G. Squeeze-and-Excitation Networks. In Proceedings of the 2018 IEEE/CVF Conference on Computer Vision and Pattern Recognition, Salt Lake City, UT, USA, 18–23 June 2018; pp. 7132–7141.
6. Xie, S.; Girshick, R.; Dollár, P.; Tu, Z.; He, K. Aggregated residual transformations for deep neural networks. In Proceedings of the IEEE Conference on Computer Vision and Pattern Recognition, Honolulu, HI, USA, 21–26 July 2017; pp. 1492–1500.
7. Szegedy, C.; Vanhoucke, V.; Ioffe, S. ; Shlens J.;Wojna, Z. *Rethinking the Inception Architecture for Computer Vision*, 2016 IEEE Conference on Computer Vision and Pattern Recognition (CVPR), **2016**, 2818–2826. doi: 10.1109/CVPR.2016.308.
8. Huang G, Liu Z, Van Der Maaten L, et al. Densely connected convolutional networks. In: Proceedings of the IEEE conference on computer vision and pattern recognition. 2017:4700-4708.
9. Tan, M.; Le, Q. Efficientnet: Rethinking model scaling for convolutional neural networks. In Proceedings of the International Conference on Machine Learning, Long Beach, CA, USA, 9–15 June 2019; pp. 6105–6114.
10. Consortium. MONAI: Medical Open Network for AI. Zenodo 2020. DOI: https://doi.org/10.5281/zenodo.5728262.
11. Forum. RSNA MICCAI Brain Tumor Radiogenomic Classification Discussion 1. Available online: https://www.kaggle.com/c/rsna-miccai-brain-tumor-radiogenomic-classification/discussion/279820 (accessed on 18 December 2021).
12. Forum. RSNA MICCAI Brain Tumor Radiogenomic Classification Discussion 2. Available online: https://www.kaggle.com/c/rsna-miccai-brain-tumor-radiogenomic-classification/discussion/279777 (accessed on 18 December 2021).
